# Transformational Leadership and Project Success: Serial Mediation of Team-Building and Teamwork

**DOI:** 10.3389/fpsyg.2021.689311

**Published:** 2021-09-07

**Authors:** Hussain Ali, Shuai Chuanmin, Mansoor Ahmed, Arshad Mahmood, Muhammad Khayyam, Anna Tikhomirova

**Affiliations:** School of Economic and Management, China University of Geosciences, Wuhan, China

**Keywords:** transformational leadership, team-building, teamwork quality, project success, information system development

## Abstract

This research investigates the impact of transformational leadership (TL) style on project success (PS) in the indirect effect of serial mediation of team-building and teamwork quality (TWQ). A quantitative research approach was used for this study. Data were gathered from 374 professional information system development (ISD) project managers in Pakistan. The hypotheses were tested using regression analysis with bootstrapping. Both team-building and teamwork independently and serially mediate the relationship between the TL and PS of the project managers, respectively. The TL style of the project manager intensifies PS with team-building practices (TBP) and TWQ. The TL boosts TWQ in terms of communication, coordination, and cohesion to achieve a successful project. The findings suggest that TL is associated with PS through serial mediation of team-building and teamwork. No research to date has used this nascent methodology to explore the association between TL and PS.

## Introduction

In most organizations using project success (PS) as a survival strategy, the ubiquitous PS phenomena will remain in the near future (Musawir et al., 2017). The PS has become a universal topic for project management researchers due to the rapid expansion and heterogeneous nature of the organizations (Albert et al., 2017). Leadership literature identifies the impact of leadership on the success and management of the project; however, there is a deficient understanding of the project-based organizations (Albert et al., 2017). In the past three decades, the researcher and practitioners are interested in the psychological relationship between employees and their organizations (Barattucci et al., 2021). Research calls to explore the underlying mechanism through which leadership styles influence information system development (ISD) projects.

Full Range Leadership Theory (FRLT) (Antonakis et al., 2003) highlighted three leadership styles; transformational, transactional, and laissez-faire (Sohmen, 2013). Laissez-faire is considered destructive in project management compared to the former two leadership styles (Skogstad et al., 2007). Transformational and transactional styles have gained particular attention in project management, and some project leaders build up a meticulous leadership behavior attempt to enhance and improve the project performance (Yang et al., 2011). The leader aims to accomplish the goal by subordinates through communication and influence, as cited by Raziq et al. (2018). Project management literature (Prabhakar, 2005; Yang et al., 2011) emphasizes some preconditions of project management and PS, such as team communication, cohesiveness, collaboration, and effective and efficient team-building (Aga et al., 2016), which are the results of good project leadership. The role of the project leader is essential in all the phases of the project, from initiation to completion and delivery of the product or service according to the expected specifications of the project stakeholder (PMI, 2013). Modern management approaches to understand the attitude of the leader, in particular, toward others (Taşçi and Titrek, 2020). The rapid change in the environment brings challenges and opportunities to cope with the project successfully and is liable for sustainable development (Huemann and Silvius, 2017). The role of the project leader is greatly signified to establish and achieve the desired goal (Shao, 2018). Project success is the ultimate goal for any organization; hence, it is an indispensable subject to project management researchers (Albert et al., 2017; Fernando et al., 2018). Complex projects are likely to be successful when experienced with a high level of team communication, collaboration, and cohesiveness (Yang et al., 2011).

The present study seeks to contribute to a better understanding of the mechanisms through which the transformational leadership (TL) behavior of the project managers influences PS. A research study called for more research to understand the relationship between TL and team performance through the use of mediators representing team processes (Gundersen et al., 2012). The government of Pakistan formulated series of policies to facilitate information technology (IT) firms to promote the export of software in the international market. The previous studies also demonstrated that project leadership is critical to PS (Scott-Young et al., 2019). Although several studies (Prabhakar, 2005; Yang et al., 2011; Sohmen, 2013; Tyssen et al., 2014; Aga et al., 2016; Raziq et al., 2018) have been conducted on project leadership style (transactional and transformational) in construction, NGO, and other industries, still there is a lack of understanding in ISD sector. The environment and the approach of the ISD projects are entirely different from other sectors due to the intangible nature of the product.

According to Antonakis et al. (2003) TL is one of the basic leadership styles of full range leadership theory (FRLT). The proposed plan is to investigate the unresolved question of previous literature with empirical evidence. This study is based on the serial mediation effect of building practices (TBP) and teamwork quality (TWQ) between TL and PS in the context of the ISD project workspace. As Pollack and Matous (2019) stated, in the project management literature, no research has been found that examined the effect of unique, repeatable team-building activities and how they affect teamwork. The study also argues that the TL style of the project manager leads to TBPs that predict teamwork quality (TWQ). The TWQ contributes to PS. The novel insights provide a serial mediation of TBP and TWQ to enhance the success of the sustainable project.

## Conceptual Framework

### Project Success

Project success is an intensively discussed topic in the project management field (Standing et al., 2006; Basten et al., 2011). Traditionally, the scope, cost, and time of the iron triangle are considered key components for the measurement of PS (PMI, 2013). Whereas, the PS evaluation also encompasses additional factors including product acceptance from the customer, stakeholder, commercialization, and future project opportunity (Cooke-Davies, 2002; Serrador and Rodney, 2014). Baccarini (1999) considered that PS is related to the outcomes of a project, such as information system or research and development products, and project management success refers to the process and performance of the project in terms of cost, time, and quality (Baccarini, 1999). DeLone and McLean defined the success of an information system (IT) as PS, in contrast to the success of the project management as cited by Petter et al. (2013).

Though there is no mutual consensus among the researcher on PS criteria in the project management literature, the work by Khang and Moe (2008) and Ika et al. (2012) are comprehensive and relevant for developmental projects. Primarily, the work done by Raziq et al. (2018) and Oh et al. (2019) is more relevant to ISD projects. An investigation proposes the following as the PS determinants: the advantages the project brings to the project organization, key partners, the project team, customer satisfaction, the accomplishment of the objectives of the project organization, and marketing potential (Ika, 2015). Given the above-mentioned determinants, comprehensively, we can describe that PS involves the following factors: project completion within the scope, time, expense, quality, customer and stakeholder satisfaction, and the achievement of the goals and objectives of the project organization.

### Transformational Leadership Style

Although the subject of leadership has been under scholarly examination for several decades, there is a shortage of empirical study in the project management context (Aga et al., 2016). Full Range Leadership Theory is the most generally accepted leadership theory, and it envelops transformational, transactional, and laissez-faire styles (Sohmen, 2013). Gundersen et al. (2012) suggested that the TL style has high importance to the project-based organization. The TL is related to solid individual identification with the leader, forming a mutual vision of things to come, and a relationship between the leader and the subordinates who are undeniably dependent on something other than the straightforward trade of remunerations for consistency (Keegan and Den Hartog, 2004). The leader is the primary source of motivation and encouragement for the subordinates to bring a positive change (Raziq et al., 2018). In the literature, there seems to be a general agreement of researchers on four dimensions of TL: idealized influence, intellectual stimulation, inspirational motivation, and individualized consideration. Idealized influence is the behavior of the leader that arouses strong emotions in the followers and establishes a deep mutual understanding. The intellectual stimulation of the leader encourages the followers to be creative and induces them to develop innovative and/or creative solutions to the problem. Inspirational motivation is expressed when a leader conveys a vision that is compelling and encouraging to followers and offers demanding tasks and elevated expectations for them. Individualized consideration describes paying attention to individual followers and their personal needs (Mittal, 2016), and provides support, encouragement, and coaching to the followers (Avolio et al., 2004; Lindgren and Packendorff, 2009).

### Team-Building Practices

In literature, TBP is considered a central part of human resource management (HRM) in project-based organizations (Turner et al., 2008). Klein et al. (2009) defined team-building as “a class of formal and informal team-level interventions that focus on improving social relations and clarifying roles, as well as solving task and interpersonal problems that affect team functioning.” There is an agreement in the literature with regard to the following four components of team-building: goal setting, role clarification, interpersonal processes, and problem-solving (Klein et al., 2009). The goal-setting strategy conveys the general goals and specific objectives of the project to the team members by defining a subtask and setting schedules. As a result, the team members who are subjected to a target setting will become active in action planning to discover ways to reach specific objectives (Aga et al., 2016). Role clarification emphasizes increased communication among team members regarding their respective roles within the team. Team members exposed to role-clarification activities are expected to achieve a better understanding of the respective roles of themselves and others and their duties within the team (Klein et al., 2009). This includes clarifying the requirements of the individual roles, team norms, and mutual responsibilities of team members (Aga et al., 2016). The interpersonal process includes conflict resolution among team members and clearing up any hidden agenda (Aga et al., 2016). Problem-solving practice stresses understanding significant challenges in group tasks to develop the aptitudes relevant to the task. This is a process in which the team members identify the problems, generate considerable and relevant data, take an interest in strategic thinking and action planning, and execute and review action plans (Aga et al., 2016).

### Teamwork Quality

The definition of teamwork refers to “the interdependent components of performance required to effectively coordinate the performance of multiple individuals.” (Salas et al., 2008a). Numerous studies have sought to assess the efficiency of teamwork. The first TWQ model developed tested the collective team-task processes and focused on the level of interaction (Hoegl and Gemuenden, 2001). Previous studies demonstrated that teamwork efficiency is measured as a second-order construct consisting of communication, coordination, cohesion, mutual support, and learning in new product development (NPD) projects (Kuthyola et al., 2017). Oh et al. (2019) used the TWQ construct for the ISD project: communication, coordination, the balance of member contribution (BMC), mutual support, effort, and cohesion. Communication considers the frequency of interaction among team members, formalization, and the free exchange of information. Coordination demands to develop a general understanding among the project team members when working on parallel subtasks, and agreement on common work-down structure, schedules, budgets, and deliverables of the project. Balance of member contribution is the ability to employ the expertise of the team members to its full potential. The contribution should reflect the specific knowledge and experience of the team members. Mutual support is the ability and willingness of the team members to assist and support each other in executing their tasks. The sub construct effort is the capacity and willingness of team members to share the workload and prioritize the activities of the group over other tasks. Cohesion is the motivation of the team members to maintain the team and to understand that the team goal is more important than individual goals.

### Research Model and Hypotheses

**Figure 1:**
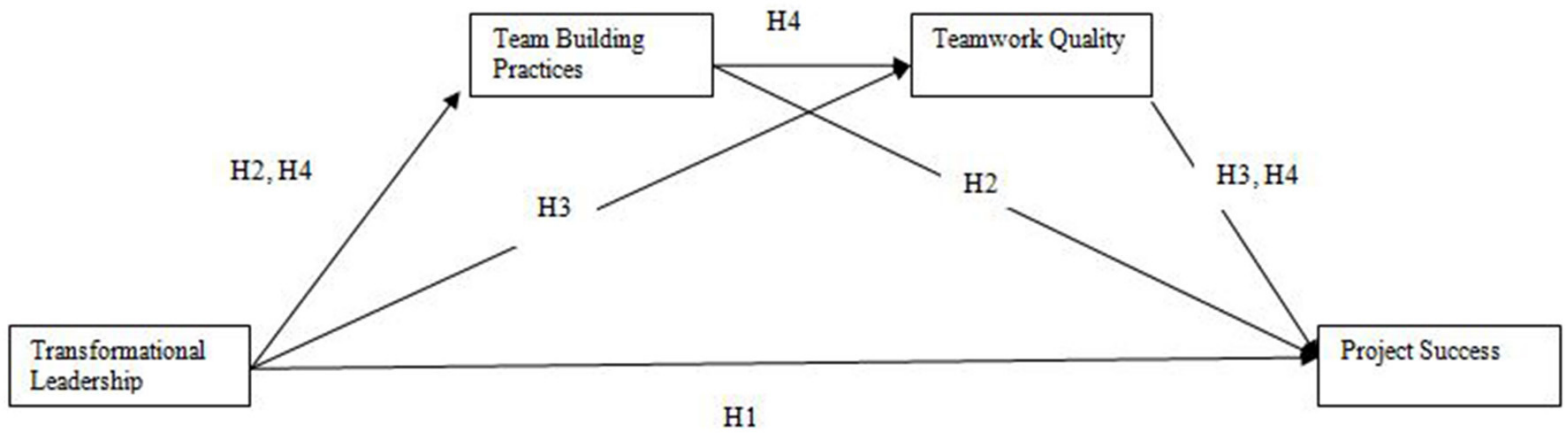
The proposed model.

This section presents the conceptual framework and hypotheses of the study. It also highlights the relationships between the variables in the study. Figure 1 depicts the conceptual framework of the study.

#### Transformational Leadership and Project Success

The literature indicates that the appropriate attitudes of the project leaders play a significant role in achieving an excellent project performance (Scott-Young and Samson, 2008; Zwikael and Unger-Aviram, 2010). The TL style is positively associated with employee entrepreneurial behavior (Afsar et al., 2017), innovative employee behavior (Wang et al., 2017), employee creativity (Dong et al., 2017), employee retention (Caillier, 2018), organizational commitment (Delegach et al., 2017), performance (Jansen et al., 2009; Vaccaro et al., 2012), employee adaptability and proactivity (Wang et al., 2017), and work engagement (Ding et al., 2017). The leadership style is also linked positively in a project environment with PS (Anantatmula, 2010) and project performance (Keller, 1992).

The attributes (inspiration, respect, obligation, individualized consideration, support, and open communication) of TL are much more likely to yield positive results (Al-Ghazali, 2020). Zaman et al. (2019) argued that TL plays a role of a catalyst in strategic repositioning and a positive change in implementing the perspective of the stakeholder, who contributes to a successful project. A transformational approach has enhanced the knowledge of different obstacles and related project problems that warrant initiatives to improve (Zaman et al., 2019). The transformational leader thus sets inspiring goals for his team members to achieve demanding project objectives. In project teams, a transformational leader promotes positive workplace relationships, high engagement, and cohesion in project teams that guarantee the success of the project (Raziq et al., 2018). In this way, the team members work to their full potential and bring the project to completion. Thus we propose our first hypothesis:

*H1. The TL style of the project manager positively influences project success*.

#### The Mediating Role of TBP

There is a strong relationship between TL style and TBP (Aga et al., 2016). Team-building facilitates the exchange of information and mutual understanding among team members and improves the outcomes of the team (Hsu et al., 2011). McDonough (2000) investigated the following four factors of the leadership style of project managers that effect TBP: project goals, empowerment, climate, and human resources. In the project goal, effective project leadership defines the goals and tasks to subordinates and clarifies the constraints. Project managers should adopt the TL style to empower team members to explore, analyze, and make decisions about the strategies to utilize them for problem-solving and tasks to be performed. Effective leadership style is vital for project managers to maintain the flow of information and expertise within the team and other organizational groups for actual decision making. This process involves communication mechanisms to exchange information about the project objectives, project changes, and updates the role and responsibilities of individual members. An effective project leadership style is required to enhance team commitment, build trust, and establish efficient working relationships among the team members. Burke et al. (2006) underlined that even if the project team is high-performing with the right skills and techniques, it will not be effective without appropriate leadership. The TL style is required for a project manager to instill motivation and enthusiasm in the team to perform beyond their expectations by conventional team-building strategies, such as goal-setting, role-clarification, interpersonal communication, and problem-solving techniques (Aga et al., 2016).

*H2. TBPs mediate the relationship between the transformational leadership style of the project manager and PS*.

#### The Mediating Role of TWQ

Several studies have indicated that teamwork is a crucial factor that positively affects the success of the project teams (Hoegl and Gemuenden, 2001; Yang et al., 2011). Teamwork is considered a proxy for project performance evaluation and project progress in complex environments (Qin et al., 2016). Several studies have shown how TWQ acts as a mediator between leadership styles and project performance (Aronson et al., 2006; Yang et al., 2011; Oh et al., 2019). Oh et al. (2019) confirmed that transformational, transactional, and entrepreneur governance network leadership styles were correlated with the TWQ and ISD proPS, and TWQ served as an essential mediator between leadership and PS. Aronson et al. (2006) analyzed TWQ as a mediator variable between the personality of the leader and project output of new product development (NPD) under different conditions and uncertainty. Another study elaborated on how TWQ mediates leadership and project outcomes (Dionne et al., 2004). In a controlled environment, the TL style enhances TWQ and leads to completion and success.

*H3. TWQ mediates the relationship between the TL style of the project manager and PS*.

#### The Serial Mediating Role of TBP and TWQ

The success of the team can have significant measurable effects on the time and cost of the success of a project; teamwork is usually seen as a soft skill with few specific guidelines or analysis of its influence on project results (Thomas et al., 2008). Team-building is one of the ways for organizations to enhance the efficiency of teamwork (Klein et al., 2009). It is an intervention in which cohesive working groups learn to improve their teamwork skills by using a planned agenda (Tannenbaum et al., 1992). Svyantek et al. (1999) investigated that team-building positively affected the efficiency of the workgroup. Klein et al. (2009) and Salas et al. (2008b) examined that team-building improves cognitive thinking (e.g., declarative knowledge of teamwork competencies), team member affective outcomes (e.g., team potency and trust), processes (e.g., coordination and communication), and team performance outcomes (e.g., productivity measures). Xu et al. (2010) found that TWQ mediates the effect of the relationship between technical and human IT infrastructure capabilities and the success of the IT project. Effective and efficient team-building, team communication, cohesiveness, and collaboration are the preconditions for the success of project management (Raziq et al., 2018). Staggers et al. (2008) strongly believed that most effective teamwork happens after team-building and team-building as a process is linked to the process of teamwork. Effective communication is an integral part of teamwork, one of every five projects being reported as unsuccessful due to poor communication (Pollack and Matous, 2019). The purpose of team-building strategies is to change and extend team processes until they are formed and stabilized (Kozlowski and Ilgen, 2006). Based on the arguments stated above, we recommend that TBPs help to enhance TWQ, which in turn will have a positive effect on PS.

*H4. TBP and TWQ serially mediate the relationship between the TL style of the project manager and PS*.

## Materials and Methods

### Research Setting and Participants

It is possible to classify projects into various categories. This research considers information systems development (ISD) projects. The deliverables of ISD projects are intangible. The development of the ISD projects aims to enhance organizational coordination in terms of real-time data, better communication, greater productivity, and a secure location. The study was designed to investigate the association between TL and PS with serial mediation of TBPs and TWQ. In this study, the participants are ISD project managers from Pakistan.

### Sample and Data Collection Procedure

The target institutions of the study were IT firms that undertake ISD projects. Data were collected in January 2020–April 2020 from ISD project managers working in leading companies in the IT sector of Pakistan. The data were collected from the registered firms with Pakistan Software Export Board (PSEB). We took the information from the firm (name, contact, website, and city) from the PSEB website. The data were gathered from the following major cities of Pakistan: Islamabad (19.0%), Peshawar (17.1%), Lahore (20.1%), Karachi (21.9%), Quetta (12.0%), and other cities (9.9%) through an online questionnaire. Some significant studies have focused on a cross-sectional approach to examine the project's success (Joslin and Müller, 2016; Musawir et al., 2017; Zaman et al., 2019). The managers have already sensed, observed, and witnessed the results of these specific characteristics of the project in question; the self-reported survey concept is more appropriate (Zhang et al., 2018). We sent the link to the online questionnaire to 800 ISD project managers. We received 374 responses; the response rate was 46.75%. In this study, we took one participant per organization. It had been mentioned at the top of the questionnaire that participants must have managed one or more ISD project(s) in the past.

### Measures

#### Project Success

In the project management literature, there is a debate on what constitutes the PS criteria. Some researchers used the aggregative approach (Yang et al., 2011; Kissi et al., 2013), whereas others used the distributive PS criteria (Dvir et al., 2003; Diallo and Thuillier, 2004). This research used the aggregate approach of measuring a multi-dimensional project performance construct based on the interpretation of specific parameters by project managers. Project success constructs adopted from a well-validated study of Aga (2016) composed of six items, addressing time, expense, quality, client use, satisfaction, and efficacy. The project managers evaluated each item on a five-point Likert scale ranging from “strongly disagree” to “strongly agree.”

#### Transformational Leadership

We adopted the constructs of Aga et al. (2016) which are based on the study by Bass and Avolio (1996). The study by Bass and Avolio (1996) has been considered a well-validated study in leadership research. We adopted eight elements of TL, covering idealized influence, inspirational motivation, intellectual stimulation, and individual consideration. Items ranging from 1 (strongly disagree) to 5 (strongly agree) were based on the five-point Likert scale, respectively.

#### Team-Building Practices and Teamwork Quality

Project TBPs and TWQ are used as serial mediator variables in the research model. Team-building is a multi-dimensional construct that entails goal setting, role clarification, interpersonal processes, and problem-solving (Klein et al., 2009). We adopted eight items from the study by Aga et al. (2016) for TBPs. The project managers assessed each item on a five-point Likert scale of 1–5 ranging between “strongly disagree” and “strongly agree,” respectively.

Teamwork quality that entails communication, coordination, the BMC, mutual support, effort, and cohesion comprises six items adapted from Oh et al. (2019) and Yang et al. (2011).

#### Covariates

In this study, the participants were ISD project managers. A set of control variables are listed that adopted to mitigate spurious effects and enhance internal validity. The control variables in this study categorize the demographics of ISD project managers. The measures of demographic control variables include gender (1 = male, 2 = female), age (1 ≤ 21 years, 2 = 21–25 years, 3 = 26–30 years, 4 = 31–35 years, 5 = 36–40 years, 6 = 41–45 years, 7 = 46–50 years, and 8 = more than 50 years), academic qualification (1 = intermediate, 2 = graduate, 3 = master/doctorate), and the experience in ISD projects (1 ≤ 1 year, 2 = 2–5 years, 3 = 6–9 years, 4 = 10–13 years, 5 = 14–17 years, and 6 = more than 17 years).

#### Data Analysis

Before analyzing the hypotheses, missing values, the accuracy of data, and outliers were examined. We used AMOS 23 and Plug-ins to conduct Confirmatory Factor Analyses (CFA) to examine convergent and discriminant validity.

We investigated the mediating effect of TBP and TWQ independently on the relationship between TL and PS. We adopted the mediation four steps methodology developed by Hayes (2013). First, the independent variable (TL) must be related to the dependent variable (PS). Second, the independent variable (TL) must be related to the mediator variable (TBPs and TWQ). Third, the dependent variable (PS) must be related to the mediator variable (TBPs and TWQ). Fourth, when the mediator variable is controlled, the effect of the independent variable (TL) on the dependent variable (PS) is no longer significant or is substantially reduced. Next, we examined the serial mediation effect of team-building and teamwork in the relationship between TL and PS.

## Results

Table 1 presents descriptive statistics of the demographics of participants. It shows 374 ISD project managers who took part in this study. In the result, 78.9% of respondents were men and 21.1% were women. The average age, experience, and education of the ISD project managers were found to be 5.47 (38 years), 4.04 (10 years), and 2.08 (graduation), respectively.

**Table 1:** Sample characteristics.

**Variables**	**Frequency (***N***)**	**Percentage**	**Average**
**Gender**			
Male	295	78.9	1.21
Female	79	21.1	
Total	374	100	
**Age**			
<21	4	1.1	5.47
21–25	6	1.6	
26–30	15	4.0	
31–35	34	9.1	
36–40	131	35	
41–45	115	30.7	
46–50	48	12.8	
>50	21	5.6	
Total	374	100	
**Experience**			
≤ 1	8	2.1	4.04
2–5	22	5.9	
6–9	88	23.5	
10–13	129	34.5	
14–17	82	21.9	
>17	45	12.0	
Total	374	100	
**Education**			
Intermediate	15	4.0	2.08
Graduation	315	84.2	
Master/Doctorate	44	11.8	
Total	374	100	

### Validity and Reliability Analyses

Convergent validity is the internal consistency of multiple dimensions for each construct. In the composite reliability (CR) statistics used for internal consistency, the recommended (Bagozzi and Yi, 1988) threshold value must be <0.70. In Table 2, the CR of TL (0.930), TBPs (0.895), TWQ (0.860), and PS (0.857) were internally consistent. Factor loadings were all >0.50.

**Table 2:** Model validity measures.

	**CR**	**AVE**	**MSV**	**MaxR(H)**	**TL**	**TBP**	**TWQ**	**PS**
TL	0.930	0.654	0.498	0.931	0.808			
TBP	0.895	0.519	0.498	0.903	0.706***	0.720		
TWQ	0.860	0.510	0.103	0.872	0.254***	0.321***	0.714	
PS	0.857	0.501	0.060	0.861	0.219***	0.245***	**–**0.030	0.708

*TL, transformational leadership; TBP, team building practices; TWQ, teamwork quality; PS, project success; CR, composite reliability; AVE, average variance extracted; MSV, maximum shared variance. Source: calculated by the authors using AMOS 23.0 and Plug-in (Gaskin and Lim, 2016)*.

Discriminant validity assesses how distinct is each construct of the model is from the others. The average variance extracted (AVE) was used to determine if the constructs had adequate discriminant validity. The acceptable threshold value for AVE should be greater than 0.50 (Fornell and Larcker, 1981). In Table 2, the results show that each construct has an acceptable AVE value: TL (0.654), TBPs (0.519), TWQ (0.510), and PS (0.501) were internally consistent. In addition, the square roots of the AVE of TL (0.808), TBPs (0.720), TWQ (0.714), and PS (0.708) were greater than the correlations between the constructs. As a result, all of the tests met the recommended convergent and discriminant validity thresholds.

The tool used for the model fit measures AMOS 23.0 and AMOS Plug-in (Gaskin and Lim, 2016). The presented results in Table 3 examined the dimensionality and fitness of the CFA model. The results indicate that our hypothesized model significantly fit the data. All the fitness indicator values are as follows: CMIN = 607.940, DF = 318, CMIN/DF = 1.912, comparative fit index (CFI) = 0.946, standard root-mean-square residual (SRMR) = 0.057, root mean square error of approximation (RMSEA) = 0.049 and PClose = 0.553 are in the acceptable range.

**Table 3:** Model fit measures.

**Measure**	**Estimate**	**Threshold**	**Interpretation**
CMIN	607.940	–	–
DF	318	–	–
CMIN/DF	1.912	Between 1 and 3	Excellent
CFI	0.946	>0.95	Acceptable
SRMR	0.057	<0.08	Excellent
RMSEA	0.049	<0.06	Excellent
PClose	0.553	>0.05	Excellent

*CMIN, contrast media-induced nephropathy; DF, degree of freedom; CFI. comparative fit index; SRMR, standardized root mean square residual; RMSEA, root mean squared error of approximation; PClose: process close. Source: calculated by the authors using AMOS 23.0 and Plug-in (Gaskin and Lim, 2016)*.

### Hypotheses Testing

**Figure 2:**
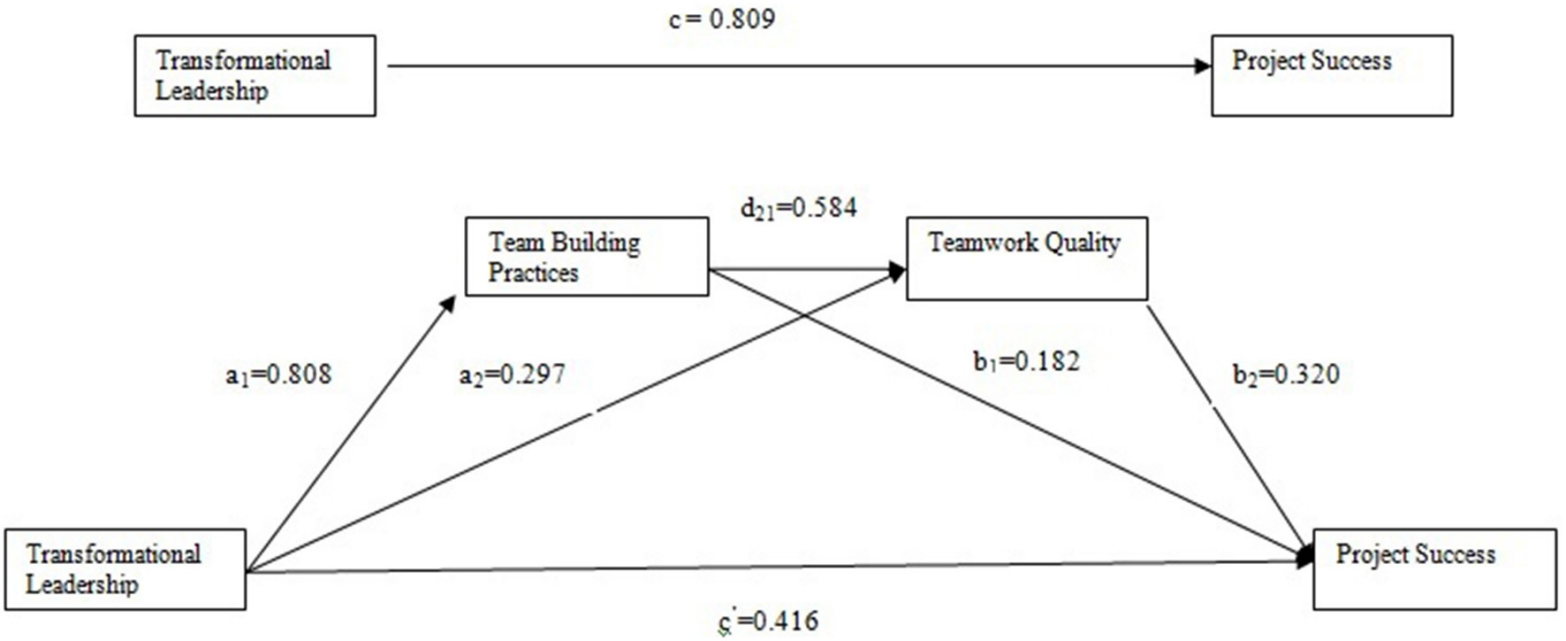
Mediation model. Notes: N = 374, *p < 0.05, **p < 0.01, ***p < 0.001.

The hypotheses were tested using an analytical method provided by Hayes (2013). The PROCESS SPSS plug-in calculated a total of 374 valid responses. Hayes (2013) argued that this method is superior to the traditional method for evaluating mediating effects. In this study, age, gender, education, and experience have considered control variables. Figure 2 shows standardized path coefficients. The proof that the 95% CI for all indirect effects does not include zero is summarized in Tables 4, 5. The results confirm that (TBPs) and TWQ mediate the relationship between TL and PS. Table 5 entailed estimates of the indirect effects along with the 98% bias-corrected bootstrapped confidence intervals for path estimates.

**Table 4:** Regression coefficients and model summary information for the serial multiple mediator model.

	**M1(TBP)**			**M2(TWQ)**			**Y(PS)**		
**Antecedent**	**Coefficient**	**SE**	***P***	**Coefficient**	**SE**	***P***	**Coefficient**	**SE**	***P***
X(TL)	a_1_0.808	0.086	<0.001	a_2_ 0.297	0.054	<0.001	c′=0.416	0.041	<0.001
M1(TBP)	–	–	–	d_21_ 0.584	0.59	<0.001	b_1_ 0.182	0.048	<0.001
M2(TWQ)	–	–	–	–			.b_2_ 0.320	0.038	<0.001
Constant i_M1_	0.522	0.086	n.s	.i_M2_−0.183	0.102	n.s	.i_y_ 0.063	0.075	n.s

*TL, transformational leadership; TBP: team building practices; TWQ: teamwork quality; PS: project success; M1: first mediator; M2: second mediator; n.s.: not significant. Source: calculated by authors by using Process in SPSS 23.0*.

**Table 5:** Indirect effects.

**Hypothesized mediating relationships**	**Indirect effect**	**BootSE**	**Lower confidence interval**	**Upper confidence interval**	**Figure path**
Ind1 → TL → TBPPS	0.147	0.060	0.280	0.516	Ind1: (a_1_ b_1)_
Ind2 TL → TWQ → PS	0.095	0.045	0.014	0.186	Ind2: (a_2_ b_2_)
Ind3 TL → TBP → TWQ → PS	0.151	0.040	0.080	0.239	Ind3: (a_1_ b_1_ +d_21_b_2_ + a_2_ b_2_)
Total indirect effects	0.393	0.060	0.280	0.516	

*Bootstrap sample 10,000 with 95% confidence interval. TL: transformational leadership; TBP: team building practices; TWQ: teamwork quality; PS: project success. Source: calculated by authors by using PROCESS in SPSS 23.0*.

Hypothesis 1 states that the TL style of the project manager positively influences PS. For H1, the analysis shows the total direct effect of TL on PS. The regression analysis indicates that TL significantly (β = 0.417, SE = 0.041, *P* < 0.000) influence PS, supporting H1. Hypothesis 2 states that TBPs mediate the relation of TL and PS. The H2 analysis requires the indirect effect of TL through TBPs to predict PS. The results shows that TBPs significantly (β = a_1_ × b_1_ = 0.807 × 0.174 = 0.140, SE = 0.073, BootLLCI = 0.018, BootULCI = 0.306) mediate the relationship between TL and PS, supporting H2. Hypothesis 3 states that the TL of the project manager predicts TWQ which affects PS. For H3 analysis, the significance test required the prediction of the indirect effect on PS of TL through TWQ. According to the result, TWQ significantly (β = a_2_ × b_2_ = 0.290 × 0.328 = 0.095, SE = 0.043, BootLLCI = 0.015, BootULCI = 0.182) mediates the relationship of TL and PS, H3 supporting H4 states that TBPs and TWQ sequentially mediate the relationship between TL and PS. For H4, the results show that TBPs and TWQ significantly (β = a_1_ × d_21_ × b_2_ = 0.807 ×0.582 × 0.328 = 0.154, SE = 0.039, BootLLCI = 0.084, BootULCI = 0.235) and serially mediate the relationship between TL and PS. The results supported all hypotheses.

## Discussion

The purpose of the present study is to empirically investigate how the TL style of the project manager influences PS directly and indirectly by multiple mediation and robust analysis. Project management necessitates the use of team-building strategies and teamwork. Gundersen et al. (2012) suggested that the TL style has high importance to the project-based organization. The following are some significant findings of the study.

First, the study shows that TL style positively influences PS. The TL style of the project manager promoted the perception of PS among the project team members. If the team members believe that the project is going in the right direction, there will be no doubt about the success. Second, there is a positive association between TL style and TBPs. The TL positively influences and clarifies the primary goal of the project, expectations, and responsibilities of individual team members and identifies task-related problems, and generates ideas, and resolves the problems. Third, the results indicate that TBPs mediate the relationship between TL and PS. Aga et al. (2016) also found a strong association among the TL style of the project manager, PS, and TBPs. Team-building practice is found to mediate the relationship between TL and PS.

Fourth, the TL style of the project manager promotes TWQ. The project team recognizes individual strengths and weaknesses and assigns work regarding the strength of the individual. The project team members are mutually helpful and supportive. Fifth, the TWQ mediates the relationship between TL and PS. Transformational leadership emphasizes that inspirational motivation, individualized consideration, and intellectual stimulation are positive effects of TWQ. In turn, TWQ increases the level of PS. Sixth, more importantly, the studies also found that TBPs and TWQ serially mediate the association between the TL style of the project manager and PS. This is the first study that explicitly identifies the serial mediation role of TBPs and TWQ in the relationship between TL and PS.

### Theoretical Implications

The current study incorporates leadership theory in project management literature in the context of the multiple mediation effect of TBPs and TWQ between TL style and PS. The study results show that TBP and TWQ link the relationship between TL and ISD of PS. The study also presents that TBPs and TWQ serially mediate the relationship between TL and ISD of PS. This finding strengthens our knowledge of the importance of TL in project management.

As our results found, TL style influences PS with or without team-building and teamwork mediation. Project management literature also found that the TL, directly and indirectly, explains the PS in the context of team-building (Aga et al., 2016) and TWQ (Yang et al., 2011). The presented results show that TL influences TBPs that contribute to TWQ, which in turn predicts the ISD of PS. In other words, TBPs and TWQ serially intervene in the path of TL and PS.

### Limitation and Future Research Direction

Our study has several limitations that should be taken into account and considered as a direction for future research.

First, the provided practical implication is limited to ISD projects. We collected data from ISD project managers in a specific country (Pakistan), which restricts the universality. Future research should focus on reassessing and reconfirming our findings in different work environments and countries.

Second, the data were collected for different constructs from one source (ISD project managers) at the same time. Therefore, a common method could be a concern. However, the results of CFA also confirm that variables are empirically distinct and the constructs used in the present study are widely applied in previous empirical research studies.

Third, we used cross-sectional data rather than longitudinal data, which are the restricting factors to draw strong inferences about causality. To better understand causality direction, future research should be conducted using longitudinal and/or experimental data sets.

Fourth, the control variable project type has not been examined in this study. The Project type in terms of team size, cost, and complexity could influence the PS. We recommend that future research should concentrate on investigating the effect of project type on PS.

The demographics of the respondents, such as age, gender, and experience need to be explored as moderators in future studies.

Since this is the first study of its kind that found team-building and TWQ serially mediate the relationship between TL and PS, respectively. We encourage the researchers to extend our model and explore more paths to PS.

## Conclusions and Implications

The purpose of the present study is to investigate the direct and indirect influence of TL style on ISD projects. The research demonstrated that TL has a direct impact on the PS of ISD. The results indicated that TBPs and TWQ serially mediate between TL and PS. In addition, we showed that team-building and TWQ independently and serially mediate the path between TL and PS. The project-based organizations should promote the TL style in project managers through leadership development programs.

This study has several practical implications. The TL style of the project managers intensifies the success of the project with TBPs and TWQ. The TL style enhances the ability of the project managers to improve their performance (Aga et al., 2016). As a result, adopting TL increases the probability of achieving the project objectives and benefiting the organizations. Team-building aims to clarify the roles, bettering social relations, and solving task-oriented problems. The TL boosts TWQ in terms of communication, coordination, and cohesion to achieve successful projects. This implies that the project managers should focus on the TL style and implement properly all components of team-building and TWQ to get a higher probability of PS. This is the responsibility of the transformational leader to create a climate to apply all ingredients of team-building and teamwork.

## Data Availability Statement

The raw data supporting the conclusions of this article will be made available by the authors, without undue reservation.

## Ethics Statement

Ethical review and approval was not required for the study on human participants in accordance with the local legislation and institutional requirements. The patients/participants provided their written informed consent to participate in this study.

## Author Contributions

SC: supervised. HA: devised the research idea and methodology. HA, AM, and SC: formal analysis. MK and HA: investigation and writing—original draft preparation. HA and AT: data curation. HA, MA, and SC: writing—review and editing. All authors contributed to the article and approved the submitted version.

## Conflict of Interest

The authors declare that the research was conducted in the absence of any commercial or financial relationships that could be construed as a potential conflict of interest.

## Publisher's Note

All claims expressed in this article are solely those of the authors and do not necessarily represent those of their affiliated organizations, or those of the publisher, the editors and the reviewers. Any product that may be evaluated in this article, or claim that may be made by its manufacturer, is not guaranteed or endorsed by the publisher.
