# Supplementary material for: Transformational Leadership and Project Success: Serial Mediation of Team-Building and Teamwork
Source: Front Psychol. 2021 Sep 7;12:689311. doi: 10.3389/fpsyg.2021.689311 (PMC8453157; doi:10.3389/fpsyg.2021.689311)
Supplement: Supplementary file 1 [file Data_Sheet_1.PDF]

Research Topic: An Empirical Study on The Role of Leadership Styles in Information System Development Projects in Pakistan

Note: This questionnaire is for those individuals who managed one or more information system development project(s)

Dear Respondent,

You have been invited to participate in the research title of “An Empirical Study on the role of leadership Styles in Information System Development Projects in Pakistan”. This survey is the part Ph.D. dissertation at China University of Geosciences, Wuhan, China. This survey will require a few minutes of your precious time.

Thank you for your participation.
Researcher: Hussain Ali
hussainali@cug.edu.cn

* Required

Demographics

1. a) Gender *

Mark only one oval per row.

	a) Male	b) Female
-	⬭	⬭

2. b) Designation *

Mark only one oval per row.

	a) Project Manager	b) Team Leader	d) Other
,	⬭	⬭	⬭

3. c) Age *

Mark only one oval per row.

	a) <21	b) 21-25	c) 26-30	d) 31-35	e) 36-40	f) 41-45	g) 46-50	h) >50
,	⬭	⬭	⬭	⬭	⬭	⬭	⬭	⬭

4. d) Education *

Mark only one oval per row.

	a) Intermediate	b) Graduate	c) Master/Doctorate
-	⬭	⬭	⬭

5. e) Experience in Software Development (in years): *

Mark only one oval per row.

	a) <=1	b) 2-5	c) 6-9	d) 10-13	e) 14-17	f) >17
-	⬭	⬭	⬭	⬭	⬭	⬭

6. g) City of your organization *

Mark only one oval per row.

	a) Islamabad	b) Peshawar	c) Lahore	d) Karachi	e) Quetta	f) Other
-	⬭	⬭	⬭	⬭	⬭	⬭

Project Success

Strongly Disagree(SD)=1 Disagree(D)=2 Neutral(N)=3 Agree(A)=4 Strongly Agree(SA)=5

7. 1. The project was completed on time. *

Mark only one oval per row.

	SD=1	D=2	N=3	A=4	SA=5
-	⬭	⬭	⬭	⬭	⬭

8. 2. The project was completed within allocated budget *

Mark only one oval per row.

	SD=1	D=2	N=3	A=4	SA=5
-	⬭	⬭	⬭	⬭	⬭

9. 3. The outcomes of the project are likely to be sustained. *

Mark only one oval per row.

	SD=1	D=2	N=3	A=4	SA=5
-	⬭	⬭	⬭	⬭	⬭

10. 4. The product (software application) met the quality needs and requirements of the end-users/customers/sponsors. *

Mark only one oval per row.

	SD=1	D=2	N=3	A=4	SA=5
-	⬭	⬭	⬭	⬭	⬭

11. 5. The outcomes of the project have directly benefited the intended end-users, either through increased efficiency or effectiveness *

Mark only one oval per row.

	SD=1	D=2	N=3	A=4	SA=5
-	⬭	⬭	⬭	⬭	⬭

12. 6. The target beneficiaries were satisfied with the outcomes of the project. *

Mark only one oval per row.

	SD=1	D=2	N=3	A=4	SA=5
-	⬭	⬭	⬭	⬭	⬭

Team-building Practices

Strongly Disagree(SD)=1 Disagree(D)=2 Neutral(N)=3 Agree(A)=4 Strongly Agree(SA)=5

13. 1. Made the basic goals of the project clear to the project team. *

Mark only one oval per row.

	SD=1	D=2	N=3	A=4	SA=5
-	⬭	⬭	⬭	⬭	⬭

14. 2. The project team received timely feedback on performance in relation to the goals of the project. *

Mark only one oval per row.

	SD=1	D=2	N=3	A=4	SA=5
-	⬭	⬭	⬭	⬭	⬭

15. 3. The information was available about share responsibilities to each team member according to the assigned role. *

Mark only one oval per row.

	SD=1	D=2	N=3	A=4	SA=5
-	⬭	⬭	⬭	⬭	⬭

16. 4. Clarified role expectations of each team member. *

Mark only one oval per row.

	SD=1	D=2	N=3	A=4	SA=5
-	⬭	⬭	⬭	⬭	⬭

17. 5. Made project norms familiar to each team member. *

Mark only one oval per row.

	SD=1	D=2	N=3	A=4	SA=5
-	⬭	⬭	⬭	⬭	⬭

18. 6. The team was discussing relationships among project members frankly to resolve conflicts. *

Mark only one oval per row.

	SD=1	D=2	N=3	A=4	SA=5
-	⬭	⬭	⬭	⬭	⬭

19. 7. The project team(s) was involved in identifying task-related problems. *

Mark only one oval per row.

	SD=1	D=2	N=3	A=4	SA=5
-	⬭	⬭	⬭	⬭	⬭

20. 8. The project team(s) was involved in generating ideas concerning the causes of task-related problems. *

Mark only one oval per row.

	SD=1	D=2	N=3	A=4	SA=5
-	⬭	⬭	⬭	⬭	⬭

Teamwork Quality

Strongly Disagree(SD)=1 Disagree(D)=2 Neutral(N)=3 Agree(A)=4
Strongly Agree(SA)=5

21. 1. There was frequent communication (meetings, phone conversations.. etc) within the team. *

Mark only one oval per row.

	SD=1	D=2	N=3	A=4	SA=5
-	⬭	⬭	⬭	⬭	⬭

22. 2. The work done on subtasks within the project was closely harmonized. *

Mark only one oval per row.

	SD=1	D=2	N=3	A=4	SA=5
-	⬭	⬭	⬭	⬭	⬭

23. 3. The team recognized the specific potentials (strengths and weaknesses) of individual team members. *

Mark only one oval per row.

	SD=1	D=2	N=3	A=4	SA=5
-	⬭	⬭	⬭	⬭	⬭

24. 4. The team members helped and supported each other as best they could. *

Mark only one oval per row.

	SD=1	D=2	N=3	A=4	SA=5
-	⬭	⬭	⬭	⬭	⬭

25. 5. Every team member fully pushed the project. *

Mark only one oval per row.

	SD=1	D=2	N=3	A=4	SA=5
-	◯	◯	◯	◯	◯

26. 6. It was important to the members of our team to be part of this project. *

Mark only one oval per row.

	SD=1	D=2	N=3	A=4	SA=5
-	◯	◯	◯	◯	◯

Goal Clarity

Strongly Disagree(SD)=1 Disagree(D)=2 Neutral(N)=3 Agree(A)=4 Strongly Agree(SA)=5

27. 1. There were clear and comprehensible goals for this project. *

Mark only one oval per row.

	SD=1	D=2	N=3	A=4	SA=5
-	◯	◯	◯	◯	◯

28. 2. The goals and requirements of the customers were clear for this project. *

Mark only one oval per row.

	SD=1	D=2	N=3	A=4	SA=5
-	◯	◯	◯	◯	◯

29. 3. The goals and requirements of the management were clear for this project. *

Mark only one oval per row.

	SD=1	D=2	N=3	A=4	SA=5
-	◯	◯	◯	◯	◯

30. 4. Substantial project goals changed during the project R *

Mark only one oval per row.

	SD=1	D=2	N=3	A=4	SA=5
-	◯	◯	◯	◯	◯

31. 5. Project goals were changed often R. *

Mark only one oval per row.

	SD=1	D=2	N=3	A=4	SA=5
-	◯	◯	◯	◯	◯

Transformational Leadership

Strongly Disagree(SD)=1 Disagree(D)=2 Neutral(N)=3 Agree(A)=4 Strongly Agree(SA)=5

32. 1. As a project manager, I talked optimistically to my team about the future. *

Mark only one oval per row.

	SD=1	D=2	N=3	A=4	SA=5
-	◯	◯	◯	◯	◯

33. 2. As a project manager, I talked enthusiastically about what needs to be accomplished *

Mark only one oval per row.

	SD=1	D=2	N=3	A=4	SA=5
-	◯	◯	◯	◯	◯

34. 3. Team members had complete faith in me. *

Mark only one oval per row.

	SD=1	D=2	N=3	A=4	SA=5
-	⬭	⬭	⬭	⬭	⬭

35. 4. As a project manager, I sought different perspectives when solving the problems *

Mark only one oval per row.

	SD=1	D=2	N=3	A=4	SA=5
-	⬭	⬭	⬭	⬭	⬭

36. 5. I provided team members with new ways of looking at puzzling things *

Mark only one oval per row.

	SD=1	D=2	N=3	A=4	SA=5
-	⬭	⬭	⬭	⬭	⬭

37. 6. I treated each team member as an individual rather than just a member of a group. *

Mark only one oval per row.

	SD=1	D=2	N=3	A=4	SA=5
-	⬭	⬭	⬭	⬭	⬭

38. 7. Being a project manager, I built each team member respect. *

Mark only one oval per row.

	SD=1	D=2	N=3	A=4	SA=5
-	⬭	⬭	⬭	⬭	⬭

Transactional Leadership

Strongly Disagree(SD)=1 Disagree(D)=2 Neutral(N)=3 Agree(A)=4 Strongly Agree(SA)=5

39. 1. I told to team members what to do if they want to be rewarded for their work. *

Mark only one oval per row.

	SD=1	D=2	N=3	A=4	SA=5
-	⬭	⬭	⬭	⬭	⬭

40. 2. I provided recognition or rewards when team members reach their goals. *

Mark only one oval per row.

	SD=1	D=2	N=3	A=4	SA=5
-	⬭	⬭	⬭	⬭	⬭

41. 3. I called attention to what team members can get for what they accomplish. *

Mark only one oval per row.

	SD=1	D=2	N=3	A=4	SA=5
-	⬭	⬭	⬭	⬭	⬭

42. 4. I concentrated my full attention on dealing with mistakes and failures. *

Mark only one oval per row.

	SD=1	D=2	N=3	A=4	SA=5
-	⬭	⬭	⬭	⬭	⬭

43. 5. I focused my attention on irregularities, exceptions, and deviations from standards *

Mark only one oval per row.

	SD=1	D=2	N=3	A=4	SA=5
-	⬭	⬭	⬭	⬭	⬭

44. 6. I closely monitored performance for errors needing correction. *

Mark only one oval per row.

	SD=1	D=2	N=3	A=4	SA=5
-	⬭	⬭	⬭	⬭	⬭

Shared Leadership

Not at All (NA)=1 To a Very Little Extent (VLE)=2 To Some Extent (SE)=3 To a Great Extent (GE)=4 To a Very Great Extent (SA)=5

45. 1. Spent time discussing our team's purpose, goals, and expectations for the project. *

Mark only one oval per row.

	SD=1	D=2	N=3	A=4	SA=5
-	⬭	⬭	⬭	⬭	⬭

46. 2. Discussed our team's main tasks and objectives to ensure that we have a fair understanding. *

Mark only one oval per row.

	SD=1	D=2	N=3	A=4	SA=5
-	⬭	⬭	⬭	⬭	⬭

47. 3. Devised action plans and time schedules that allow for meeting our team's goals. *

Mark only one oval per row.

	SD=1	D=2	N=3	A=4	SA=5
-	⬭	⬭	⬭	⬭	⬭

48. 4. Recognized each other's accomplishments and hard work. *

Mark only one oval per row.

	SD=1	D=2	N=3	A=4	SA=5
-	⬭	⬭	⬭	⬭	⬭

49. 5. Encouraged team members who seem frustrated. *

Mark only one oval per row.

	SD=1	D=2	N=3	A=4	SA=5
-	⬭	⬭	⬭	⬭	⬭

50. 6. People were encouraged to speak up to test assumptions about issues under discussion. *

Mark only one oval per row.

	SD=1	D=2	N=3	A=4	SA=5
-	⬭	⬭	⬭	⬭	⬭

51. 7. Everyone on this team has a chance to participate and provide input. *

Mark only one oval per row.

	SD=1	D=2	N=3	A=4	SA=5
-	⬭	⬭	⬭	⬭	⬭

52. 8. My team supported everyone actively participating in decision making. *

Mark only one oval per row.

	SD=1	D=2	N=3	A=4	SA=5
-					

This content is neither created nor endorsed by Google.

Google Forms
